# C-MYC-activated lncRNA SNHG20 accelerates the proliferation of diffuse large B cell lymphoma via USP14-mediated deubiquitination of β-catenin

**DOI:** 10.1186/s13062-024-00488-9

**Published:** 2024-06-18

**Authors:** Chaoyu Wang, Wen Fu, Youju Zhang, Xiaoge Hu, Qiuran Xu, Xiangmin Tong

**Affiliations:** 1 College of Biotechnology and Bioengineering, Zhejiang University of Technology, Hangzhou, 310000 China; 2 Zhejiang Key Laboratory of Tumor Molecular Diagnosis and Individualized Medicine, Zhejiang Provincial People’s Hospital, Affiliated People’s Hospital, Hangzhou Medical College, Hangzhou, 310014 China; 3 Department of Hepatobiliary Surgery, The First Affiliated Hospital of Xi’an Jiaotong University, Xi’an, 710061 China; 4 Department of Hepatobiliary and Pancreatic Surgery, Zhejiang Provincial People’s Hospital, Affiliated People’s Hospital, Hangzhou Medical College, Hangzhou, 310014 China; 5 Department of Clinical Research Center, Affiliated Hangzhou First People’s Hospital, School of Medicine, Westlake University, Hangzhou, 310003 China

**Keywords:** DLBCL, SNHG20, c-MYC, USP14, Β-catenin

## Abstract

**Background:**

Long noncoding RNAs (lncRNAs) are implicated in the initiation and progression of diffuse large B-cell lymphoma (DLBCL). Small nucleolar RNA host gene 20 (SNHG20) has been recognized as a critical lncRNA in multiple human cancers. However, the role of SNHG20 and its underlying mechanism in DLBCL are still unclear.

**Methods:**

The expression levels of SNHG20, c-MYC, β-catenin, and ubiquitin-specific peptidase 14 (USP14) were measured by reverse transcription-quantitative polymerase chain reaction (RT‒qPCR) and immunoblotting. Cell Counting Kit-8 (CCK-8), 5-Ethynyl-2′-deoxyuridine (EdU) incorporation, and flow cytometry assays were used to assess the proliferation and apoptosis of DLBCL cells. The transcriptional regulation of SNHG20 by c-MYC was confirmed by a luciferase reporter assay and RNA immunoprecipitation. The interaction between USP14 and β-catenin was demonstrated using coimmunoprecipitation. A subcutaneous xenograft model was constructed to determine the role of SNHG20 in vivo.

**Results:**

In the present study, we found that SNHG20 expression was upregulated in DLBCL cell lines and tissues compared to their normal counterparts. SNHG20 knockdown prominently reduced the proliferation and induced the apoptosis of U2932 and OCI-LY3 cells. However, SNHG20 overexpression increased the proliferation and apoptosis resistance of DLBCL cells. Mechanistically, the expression of SNHG20 was positively regulated by c-MYC in DLBCL cells. C-MYC directly bound to the promoter of SNHG20 to activate its transcription. SNHG20 was expressed mainly in the cytosol in DLBCL cells. SNHG20 silencing did not impact USP14 expression but markedly decreased the level of β-catenin, the substrate of USP14, in DLBCL cells. USP14 overexpression increased the β-catenin level, and this increase was attenuated by SNHG20 knockdown. Treatment with the proteasome inhibitor MG132 abolished SNHG20 knockdown-induced β-catenin downregulation. Moreover, SNHG20 silencing reduced the half-life but increased the ubiquitination of β-catenin in DLBCL cells. SNHG20 knockdown weakened the interaction between both endogenous and exogenous USP14 and β-catenin. In turn, SNHG20 overexpression increased the c-MYC level, and this increase was attenuated by β-catenin knockdown. Importantly, β-catenin knockdown attenuated the SNHG20-mediated increase in DLBCL cell proliferation in vitro and tumour growth in vivo.

**Conclusions:**

Taken together, our results suggested that c-MYC-activated SNHG20 accelerated the proliferation and increased the apoptosis resistance of DLBCL cells via USP14-mediated deubiquitination of β-catenin. The c-MYC/SNHG20 positive feedback loop may be a new target for anti-DLBCL treatment.

**Supplementary Information:**

The online version contains supplementary material available at 10.1186/s13062-024-00488-9.

## Introduction

Diffuse large B-cell lymphoma (DLBCL) is an aggressive type of B-cell lymphoma [1]. Currently, the standard therapeutic strategy for DLBCL is CHOP (cyclophosphamide, doxorubicin, vincristine, prednisone) chemotherapy plus rituximab [2], which can achieve a cure in 60–70% of patients. However, a portion of patients experience drug resistance have poor clinical outcomes [3]. Hence, gaining a thorough understanding of tumorigenesis in DLBCL is crucial.

Long noncoding RNAs (lncRNAs) are a class of noncoding RNAs with a length of greater than 200 nt and little protein-coding capacity. It has been proven that lncRNAs regulate tumour progression in many cancers, including haematologic malignancies [4, 5]. For instance, the lncRNA small nucleolar RNA host gene 16 (SNHG16) plays an oncogenic role by promoting cell cycle progression and suppressing apoptosis in multiple myeloma cells [6]. The lncRNA LINC01268 is overexpressed in acute myeloid leukaemia (AML) and promotes AML progression in vitro [7]. In DLBCL, the lncRNA noncoding RNA activated by DNA damage (NORAD) promotes disease progression by activating the PI3K/AKT pathway [8]. In recent studies, small nucleolar RNA host gene 20 (SNHG20) was found to play oncogenic roles in many tumours [9]. SNHG20 contributes to the proliferation and migration of lung cancer cells by acting as an epigenetic regulator of P21 [10]. SNHG20, which is stabilized by zinc finger RANBP2-type containing 2 (ZRANB2), maintains the stability of forkhead box K1 (FOXK1) mRNA to accelerate glioma progression [11]. However, the biological role and potential molecular mechanisms of SNHG20 in DLBCL have never been reported.

c-MYC has been shown to function as a transcription factor and to be aberrantly expressed in DLBCL [12, 13]. Previous studies have demonstrated that c-MYC is involved in many cellular processes, including cell proliferation [14], drug resistance [15] and aerobic glycolysis [16]. To date, c-MYC has been found to be involved in the regulation of the expression of various lncRNAs and thus influence disease progression in haematological malignancies [17]. For example, c-MYC regulates the expression of the lncRNA small nucleolar RNA host gene 12 (SNHG12) to modulate cell proliferation as well as drug resistance in natural killer/T-cell lymphoma [18]. In DLBCL, c-MYC activates the expression of the lncRNA nuclear paraspeckle assembly transcript 1 (NEAT1) and functional intergenic repeating RNA element (FIRRE), which influence cell proliferation and apoptosis [14, 19]. However, it is still unknown whether c-MYC participates in the regulation of SNHG20 expression in DLBCL.

Here, we assessed the level of SNHG20 in DLBCL cell lines and tissues. The impact of SNHG20 on DLBCL cell proliferation and apoptosis was investigated. Then, the upstream and downstream regulatory mechanisms of SNHG20 were explored. We found that SNHG20 was highly expressed in DLBCL cells and tissues. Further functional experiments revealed that SNHG20 plays an oncogenic role by accelerating the proliferation and increasing the apoptosis resistance of DLBCL cells. SNHG20, which is transcriptionally activated by c-MYC, interacts with ubiquitin-specific peptidase 14 (USP14) to deubiquitinate and stabilize β-catenin. Our findings may provide a new therapeutic strategy for curing DLBCL.

## Materials and methods

### Tissue samples

We obtained forty-eight DLBCL tissue samples and twenty-four reactive lymphoid hyperplasia (RLH) tissue samples from patients at Zhejiang Provincial People’s Hospital with the approval of the Ethics Committee of Zhejiang Provincial People’s Hospital and the written informed consent of all enrolled patients. Patients with both DLBCL and other tumours were excluded. All tissues were pathologically confirmed according to the “Guidelines for the Diagnosis and Treatment of Diffuse Large B-Cell Lymphoma in China” (2022 edition).

### Cell culture

Human DLBCL cell lines, including OCI-LY10, OCI-LY8, OCI-LY3, and U2932, were previously preserved in Zhejiang Key Laboratory of Tumor Molecular Diagnosis and Individualized Medicine. The human B lymphocyte line GM12878 was obtained from FuHeng Cell Center (FH1258, Shanghai, China). The cells were cultured in RPMI-1640 medium (Vivacell, Shanghai, China) supplemented with 15% fetal bovine serum (Vivacell) and 1% penicillin/streptomycin in an atmosphere of 5% CO_2_ at 37 °C.

### Plasmids and cell transfection

The Flag-USP14 and HA-β-catenin plasmids were designed and provided by GeneChem (Shanghai, China). DLBCL cells were infected with lentiviruses containing a short hairpin RNA targeting SNHG20 (shSNHG20-1 and shSNHG20-2), c-MYC (shMYC-1 and shMYC-2), or β-catenin (shCTNNB1) or a negative scrambled control shRNA (shNT) (GenePharma, Shanghai, China) at an MOI of 50. We added polybrene (8 µg/ml) to increase the infection efficiency. The SNHG20 overexpression and negative control (EV) lentiviruses were obtained from GeneChem (Shanghai, China). We added GeneChem’s transfection reagent (received as a gift) according to the instructions to increase the transduction efficiency. For c-MYC and USP14 overexpression, we purchased lentiviruses from Genomeditech (Shanghai, China).

### Reverse transcription-quantitative PCR (RT‒qPCR)

Cytoplasmic and nuclear RNAs of DLBCL cells were extracted and purified using PARIS™ Kit (Invitrogen, Thermo Fisher Scientific, Waltham, MA, USA) according to the manufacturer’s instructions. RT‒qPCR was performed as described previously [20]. GAPDH was used as the internal control. The sequences of the primers used are listed in Supplementary Table 1.

### Western blotting

Western blotting was performed as previously described [20]. The primary antibodies used were anti‑USP14 (1:2000, Cat No. 14517-1-AP, ProteinTech, Wuhan, China), anti‑beta-catenin (1:2000, Cat No. 51067-2-AP, ProteinTech), anti‑c-MYC (1:2000, Cat No. 10828-1-AP, ProteinTech), anti-GAPDH (1:5000, Cat No. 60004-1-Ig, ProteinTech) and anti‑Vinculin (1:5000, Cat No. 66305-1-Ig, ProteinTech) antibodies. The secondary antibodies are HRP-labeled Goat Anti-Rabbit IgG (1:1000, A0208, Beyotime, Shanghai, China) and HRP-labeled Goat Anti-Mouse IgG (1:1000, A0216, Beyotime).

### Cell counting kit 8 (CCK-8) assay

Cells were seeded in a 96-well plate (15,000 cells per well in 200 µl of complete RPMI-1640 medium). Then, 20 µl of CCK-8 solution (Cat# 40203ES76, Yeasen, Shanghai, China) per well was added to the cells at the 0, 24, 48, and 72 h time points. After incubation for 3 h at 37 °C, the absorbance at 450 nm was measured using a microplate reader.

### 5-Ethynyl-2′-deoxyuridine (EdU) incorporation assay

Virus-infected DLBCL cells were seeded in a 6-well plate. EdU incorporation assays were performed using a Click-iT™ Plus EdU Cell Proliferation Kit (Thermo Fisher Scientific, Waltham, MA, USA) according to the manufacturer’s instructions.

### Apoptosis assay

A PE Annexin V Apoptosis Detection Kit I (BD Biosciences, Beijing, China) was used to evaluate apoptosis. DLBCL cells in a 1.5 mL collection tube were centrifuged at 1000 rpm for 5 min and washed with precooled PBS. Then, the cells were resuspended in 1 × binding buffer (1 × 10^6^ cells/mL). Then, 100 µL of the suspension (1 × 10^5^ cells) was transferred to a 5 mL culture tube, and PE Annexin V (5 µL) and 7-AAD (5 µL) were added. After gentle vortexing, the tubes were incubated in the dark for 15 min at room temperature. Then, 400 µL of 1 × binding buffer was added to each tube, and apoptosis was analysed by flow cytometry according to the experimental procedure. Subsequently, apoptosis data were analysed using software. Flow cytometric analysis of apoptosis was performed with DLBCL cells that were transduced with the corresponding vectors after treatment with doxorubicin (DOX; Selleck Chemicals, Houston, TX, USA) for 24 h.

### Chromatin immunoprecipitation (ChIP) assay

DLBCL cells were incubated in culture medium containing 1% formaldehyde for crosslinking for 10 min. Crosslinking was subsequently terminated by incubation with 0.125 M glycine for a 5-minute period. The cells were then lysed in ChIP lysis buffer containing 50 mM HEPES and 0.1% SDS on ice for 20 min. Sonication was performed for 10 min, and the supernatant was collected after centrifugation. The products of sonication were incubated with a monoclonal antibody against c-Myc (3 µg, MA1-980, Thermo Fisher Scientific) at 4 °C overnight for the immunoprecipitation of chromatin fragments. The immunoprecipitates were then mixed with Pierce™ Protein G agarose (20,421, Thermo Fisher Scientific) for a 2 h incubation at 4 °C. DNA was eluted utilizing elution buffer (1% SDS, 100 mM NaHCO_3_) and purified via RNase A (CW0601S, CWBIO, Beijing, China) and proteinase K (ST533, Beyotime, Shanghai, China) treatment before PCR analysis.

### Luciferase reporter assay

The Dual Luciferase Reporter Gene Assay Kit (RG027, Beyotime, Shanghai, China) was used. A vector (pGL3) containing the promoter of SNHG20 was cotransfected with another vector into HEK293T cells. Forty-eight hours after transfection, the cells were lysed and centrifuged, and the luciferase activity in the supernatant was sequentially assessed utilizing firefly and Renilla luciferase detection solution. The mean ratio of firefly luciferase to Renilla luciferase was calculated for normalization for a quantitative comparison.

### RNA binding protein immunoprecipitation (RIP) assay

RIP was performed with the Magna RIP™ RNA-Binding Protein Immunoprecipitation Kit (17–700, Millipore Sigma, Burlington, MA, USA) in accordance with the manufacturer’s protocol. Normal rabbit IgG (#2729) and an anti-USP14 primary antibody (1:100, #11,931) were purchased from Cell Signaling Technology (Danvers, MA, USA) and used for this assay.

### Coimmunoprecipitation (co-IP) assay

The interaction between USP14 and β-catenin was confirmed by co-IP with a Dynabeads™ Protein G Immunoprecipitation Kit (10007D, Thermo Fisher Scientific) in accordance with the manufacturer’s protocol. IP lysis buffer was used to extract proteins from DLBCL cells. The cell lysates were incubated with an anti-HA (1:50, #3724, Cell Signaling Technology) or anti-β-catenin (1:50, #2677, Cell Signaling Technology) primary antibody and 30 µL of a slurry of Protein G Sepharose at 4 °C overnight for immunoprecipitation of the RNA binding proteins. Immunoprecipitates were subjected to western blotting using antibodies against Flag (1:1000, #8146, Cell Signaling Technology), USP14 (1:2000, Cat No. 14517-1-AP, ProteinTech) and Ub (1:1000, #20,326, Cell Signaling Technology).

### In vivo **experiments**

Female BALB/c nude mice (Shanghai SLAC Laboratory Animal Co., Ltd, 4–6 weeks old) were utilized for assessing the in vivo growth of DLBCL. A total of 5 × 10^6^ OCI-LY3 cells (mixed with Matrigel at a 1:1 ratio) stably transduced with the indicated vectors were subcutaneously injected into the nude mice (*n* = 4 mice in each group). Tumour size was measured with a digital calliper every three days. Three weeks after subcutaneous injection of DLBCL cells, the mice were euthanized by carbon dioxide inhalation, and tumour growth curves were generated. The animal studies were approved by the Institutional Animal Care and Use Committee of Xi’an Jiaotong University.

### Statistical analysis

The experiments were repeated at least three times, and the data were analysed with GraphPad Prism 9 (GraphPad Inc., San Diego, CA, USA). In this study, continuous variables are presented as the means ± standard deviations (SDs). Student’s t test was used to estimate the significance of differences between two groups. One-way ANOVA was used to assess the significance of differences among more than two groups. A *P* value less than 0.05 was considered to indicate statistical significance.

## Results

### The expression of SNHG20 is upregulated in DLBCL tissues and cell lines

**Fig. 1:**
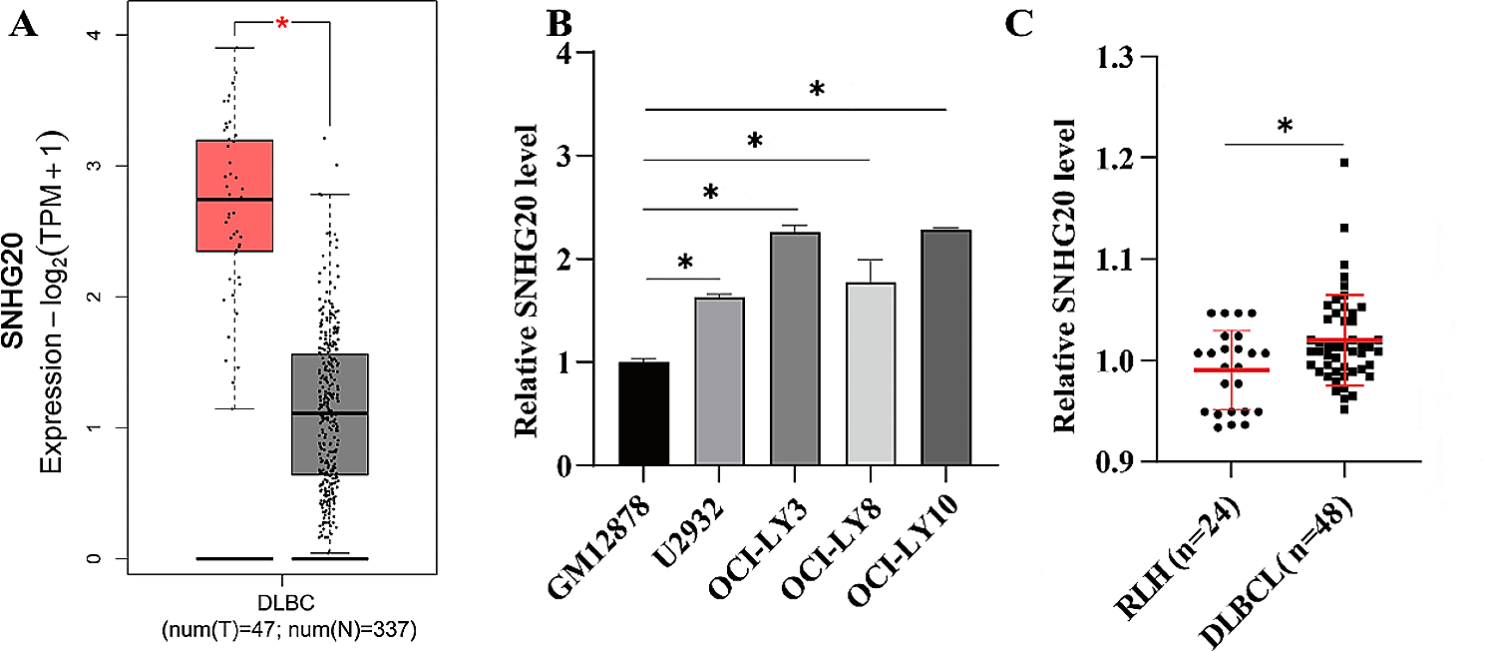
SNHG20 is upregulated in DLBCL tissues and cell lines. (**A**) Analysis of TCGA and GTEx data using the GEPIA2 platform indicated that SNHG20 was more highly expressed in DLBCL tissues (T, red) than in normal tissues (N, grey). (**B**) RT‒qPCR analysis of SNHG20 mRNA expression in DLBCL cell lines (U2932, OCI-LY3, OCI-LY8, and OCI-LY10) and human B lymphocytes (GM12878). (**C**) Quantitative analysis of RT‒qPCR data revealed that the SNHG20 expression levels in DLBCL samples (*n* = 48) were higher than those in RLH tissues (*n* = 24). *P < 0.05

The expression of SNHG20 was analysed via the Gene Expression Profiling Interactive Analysis 2 (GEPIA2) platform (http://gepia2.cancer-pku.cn/#index) [21], and we discovered that SNHG20 expression was significantly greater in DLBCL tissues than in their normal counterparts (*P* < 0.05, Fig. 1A). Then, we measured the SNHG20 level in the normal B-cell line GM12878 and DLBCL cell lines. The level of SNHG20 in DLBCL cell lines was markedly higher than that in GM12878 cells (*P* < 0.05, Fig. 1B). Moreover, DLBCL tissues and RLH tissues were collected, and RT‒qPCR analysis revealed that SNHG20 expression was markedly greater in DLBCL tissues than in RLH tissues (*P* < 0.05, Fig. 1C). Thus, the above results confirmed that SNHG20 was upregulated in DLBCL tissues and cell lines, indicating that SNHG20 may play a tumour-promoting role in DLBCL.

### SNHG20 facilitates the proliferation and suppresses the apoptosis of DLBCL cells

**Fig. 2:**
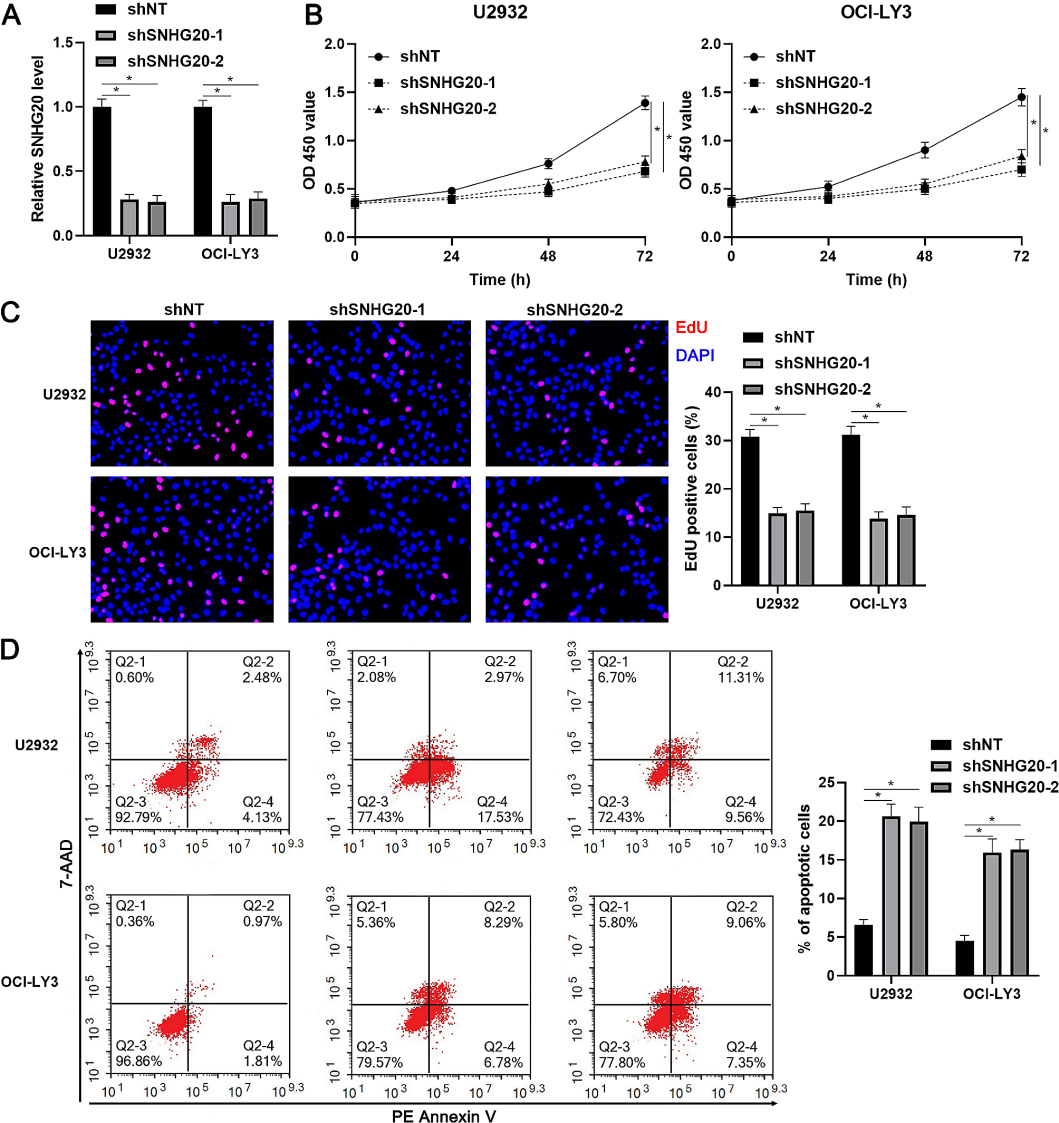
SNHG20 knockdown inhibits the proliferation and induces the apoptosis of DLBCL cells. (**A**) U2932 and OCI-LY3 cells that were transduced with a nontargeting shRNA (shNT) or shRNAs against SNHG20 (shSNHG20-1 and shSNHG20-2) were subjected to RT‒qPCR for analysis of SNHG20 expression. (**B**) The viability of DLBCL cells with or without SNHG20 knockdown was evaluated by using a CCK-8 assay. (**C**) The proliferation of DLBCL cells with or without SNHG20 knockdown was examined by using an EdU incorporation assay. (**D**) Differences in apoptosis were determined by using flow cytometry in DLBCL cells with or without SNHG20 knockdown. *P < 0.05

**Fig. 3:**
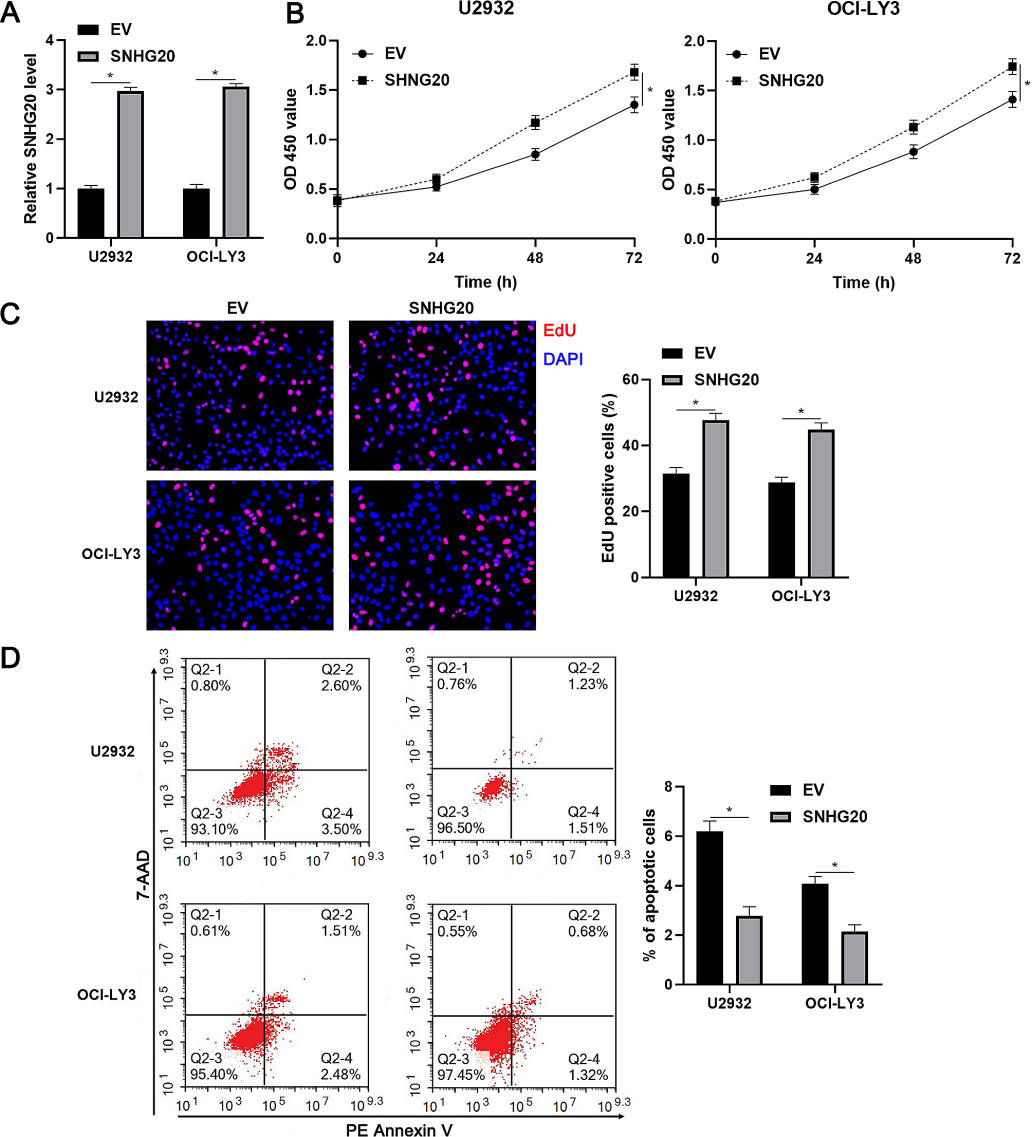
SNHG20 overexpression facilitates DLBCL cell proliferation and apoptosis resistance. (**A**) U2932 and OCI-LY3 cells that were transduced with empty vector (EV) or a vector expressing SNHG20 (SNHG20) were subjected to RT‒qPCR for analysis of SNHG20 expression. (**B**) The viability of DLBCL cells with or without SNHG20 overexpression was evaluated by using a CCK-8 assay. (**C**) The proliferation of DLBCL cells with or without SNHG20 overexpression was examined by using an EdU incorporation assay. (**D**) Differences in apoptosis were determined by using flow cytometry in DLBCL cells with or without SNHG20 overexpression. *P < 0.05

To explore the function of SNHG20 in DLBCL, we reduced SNHG20 expression in U2932 and OCI-LY3 cells by lentiviral transduction (*P* < 0.05, Fig. 2A). The proliferation ability of DLBCL cells was markedly weakened by SNHG20 knockdown (*P* < 0.05, Fig. 2B and C). In addition, SNHG20 silencing significantly increased apoptosis in U2932 and OCI-LY3 cells (*P* < 0.05, Fig. 2D). Moreover, SNHG20 was prominently overexpressed in U2932 and OCI-LY3 cells (*P* < 0.05, Fig. 3A). Gain-of-function experiments indicated that SNHG20 overexpression markedly increased the proliferation and apoptosis resistance of DLBCL cells (*P* < 0.05, Fig. 3B and D). Moreover, SNHG20 knockdown increased but SNHG20 overexpression attenuated DOX-induced apoptosis in DLBCL cells (Supplementary Fig. 1). Therefore, the above results suggest that SNHG20 acts as an oncogene in DLBCL.

### C-MYC transcriptionally activates SNHG20 in DLBCL

**Fig. 4:**
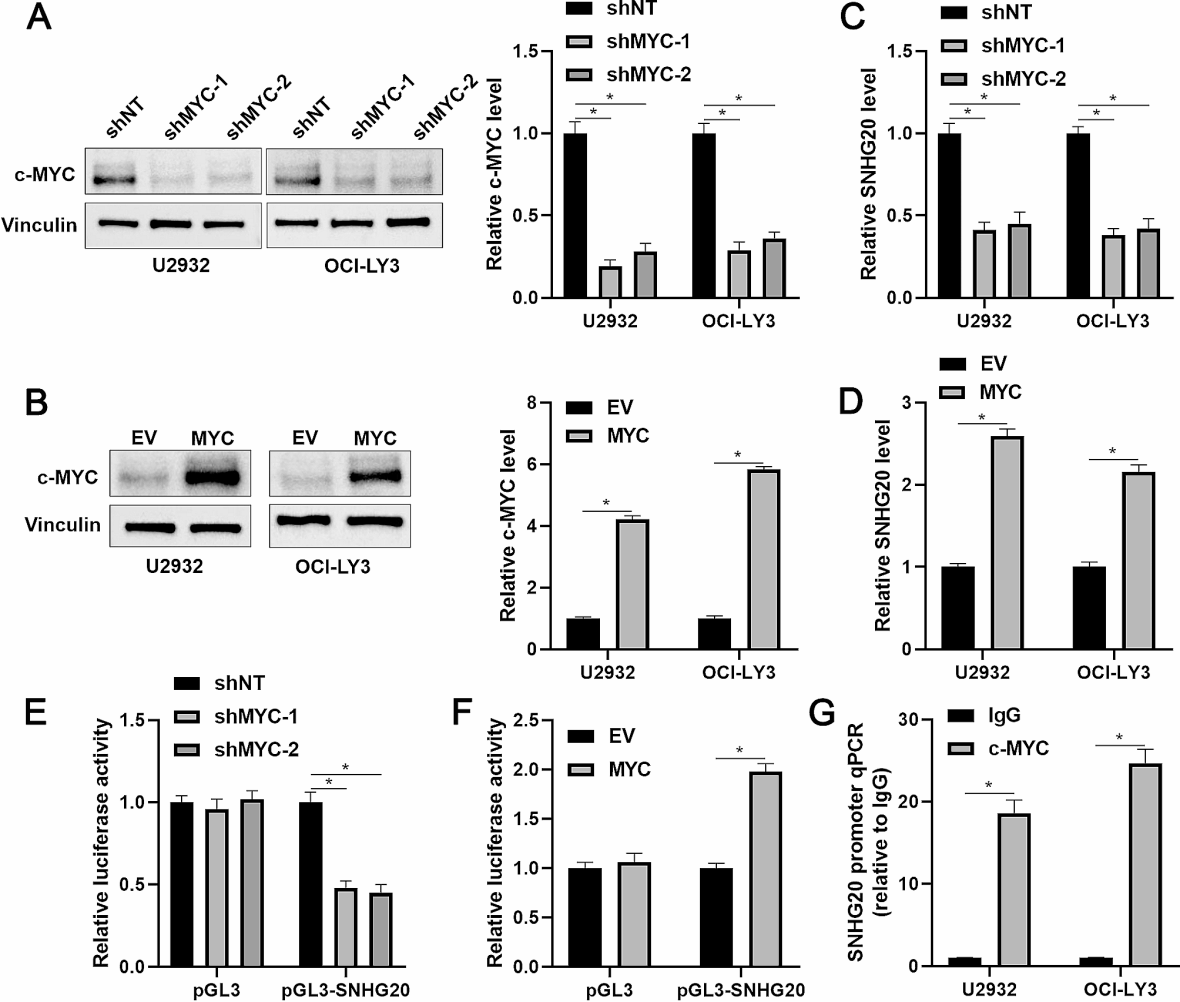
SNHG20 expression is positively regulated by c-MYC in DLBCL cells. (**A**) U2932 and OCI-LY3 cells transduced with a nontargeting shRNA (shNT) or shRNAs against c-MYC (shMYC-1 and shMYC-2) were subjected to immunoblotting for analysis of c-MYC expression. (**B**) U2932 and OCI-LY3 cells that were transduced with empty vector (EV) or a vector expressing MYC (MYC) were subjected to immunoblotting for analysis of c-MYC expression. (**C**) Quantitative analysis of RT‒qPCR data for SNHG20 expression in DLBCL cells with or without c-MYC knockdown. (**D**) Quantitative analysis of RT‒qPCR data for SNHG20 expression in DLBCL cells with or without c-MYC overexpression. (**E**) HEK293T cells were transfected with a luciferase vector containing the SNHG20 promoter (pGL3-SNHG20) or an empty vector (pGL3). Luciferase activity was measured in HEK293T cells with or without c-MYC knockdown. (**F**) Luciferase activity was measured in HEK293T cells with or without c-MYC overexpression. (**G**) Immunoprecipitation was performed using an anti-c-MYC antibody or IgG, and the SNHG20 promoter sequence was detected using RT‒qPCR. *P < 0.05

Next, we aimed to explore the underlying mechanism of SNHG20 overexpression in DLBCL. Using the JASPAR (http://jaspar.genereg.net/) database, we found that the promoter of SNHG20 contains a c-MYC binding motif (CACGTG). According to analysis via the GEPIA2 platform [21], c-MYC expression is high and positively correlated with SNHG20 expression in DLBCL tissues (*P* < 0.05, Supplementary Fig. 2). Moreover, c-MYC expression was altered via lentiviral transduction in U2932 and OCI-LY3 cells (*P* < 0.05, Fig. 4A and B). c-MYC knockdown reduced SNHG20 expression but c-MYC overexpression increased SNHG20 expression in DLBCL cells (*P* < 0.05, Fig. 4C and D). A luciferase reporter vector containing the promoter of SNHG20 was constructed. We confirmed that c-MYC positively regulated the transcriptional activity of the SNHG20 promoter (*P* < 0.05, Fig. 4E and F). The ChIP assay demonstrated that c-MYC directly bound to the SNHG20 promoter in DLBCL cells (*P* < 0.05, Fig. 4G). Thus, we identified c-MYC as a transcription factor of SNHG20 in DLBCL.

### SNHG20 participates in USP14-mediated β-catenin stability

**Fig. 5:**
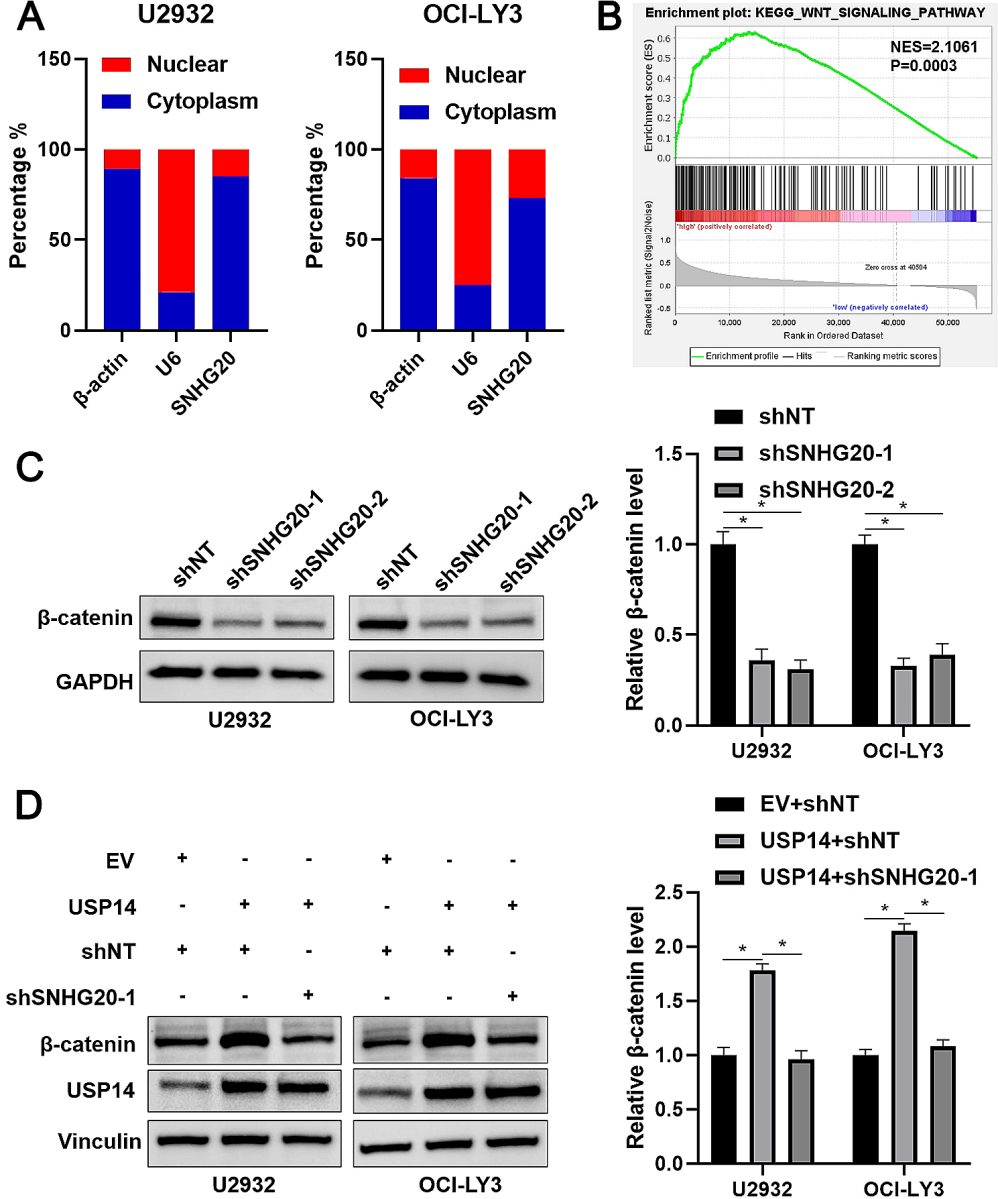
SNHG20 regulates the β-catenin abundance in DLBCL cells. (**A**) Total RNA was isolated from the nucleus and cytoplasm of U2932 and OCI-LY3 cells and subjected to RT‒qPCR for analysis of SNHG20, β-actin and U6 expression. (**B**) GSEA of the differentially expressed genes between SNHG20-high and SNHG-low DLBCL tissues represented in the TCGA database. (**C**) U2932 and OCI-LY3 cells that were transduced with a nontargeting shRNA (shNT) or shRNAs against SNHG20 (shSNHG20-1 and shSNHG20-2) were subjected to immunoblotting for analysis of β-catenin expression. (**D**) U2932 and OCI-LY3 cells overexpressing USP14 were transduced with shNT or shSNHG20-1. The protein levels of β-catenin and USP14 were measured via western blotting. *P < 0.05

**Fig. 6:**
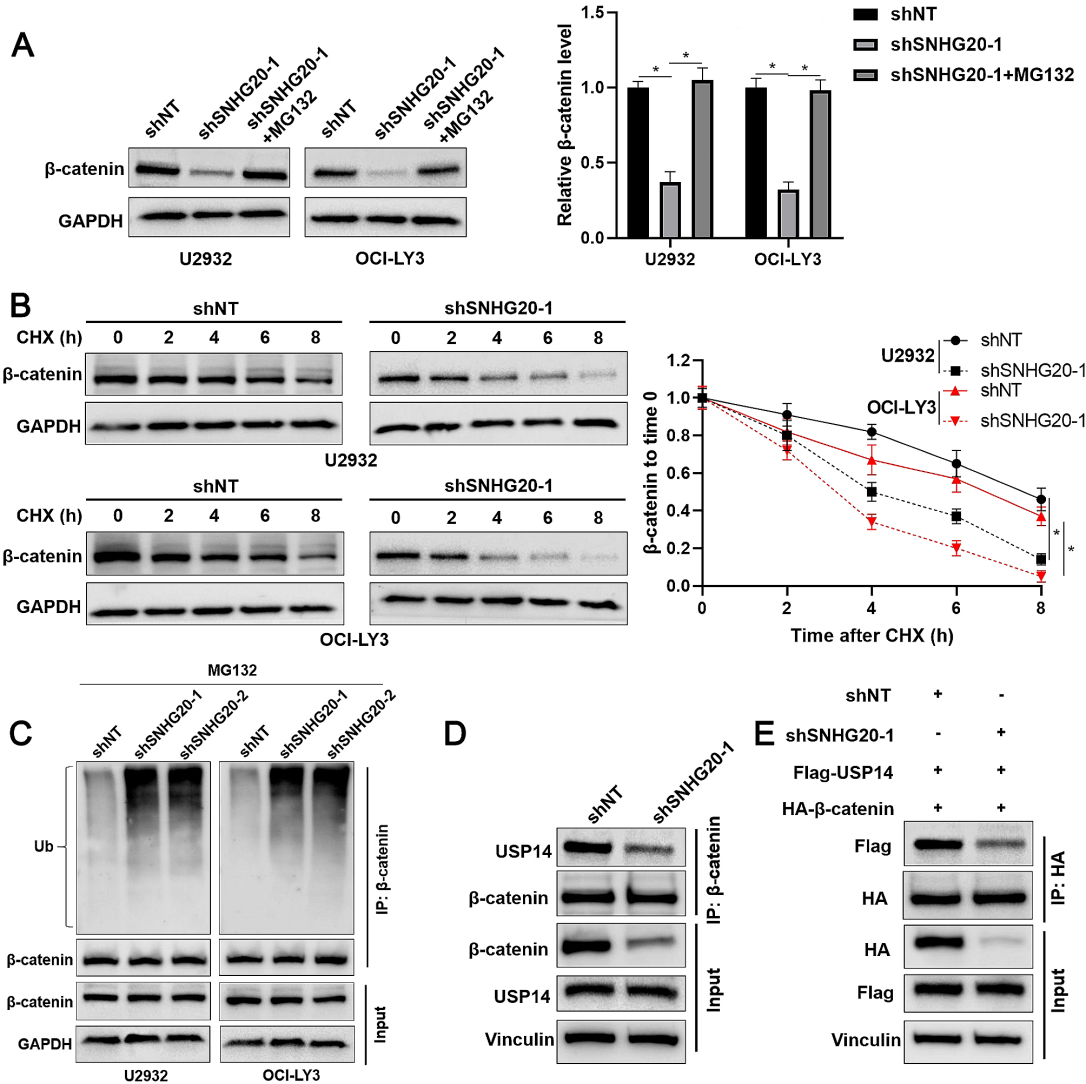
SNHG20 regulates the ubiquitination and stability of β-catenin. (**A**) U2932 and OCI-LY3 cells with SNHG20 knockdown were treated with MG132 (10 µM). The protein level of β-catenin was measured using western blotting. (**B**) U2932 and OCI-LY3 cells with or without SNHG20 knockdown were treated with CHX (40 µg/mL). The protein level of β-catenin was measured using western blotting at different time points. (**C**) U2932 and OCI-LY3 cells were transduced with shNT or shSNHG20. Immunoprecipitation was performed using an anti-β-catenin antibody, and the protein levels of Ub and β-catenin were measured via western blotting. (**D**) U2932 cells were transduced with shNT or shSNHG20. Immunoprecipitation was performed using an anti-β-catenin antibody, and the protein levels of USP14 and β-catenin were measured via western blotting. (E) HEK293T cells were transfected with the corresponding vectors. Immunoprecipitation was performed using an anti-HA antibody, and the protein levels of Flag-USP14 and HA-β-catenin were measured via western blotting. *P < 0.05

We further investigated the mechanism involved in the DLBCL-promoting role of SNHG20. qRT‒PCR analysis of nuclear and cytoplasmic extracts indicated that SNHG20 was expressed mainly in the cytoplasm of DLBCL cells (Fig. 5A). KEGG pathway enrichment analysis indicated a close correlation between SNHG20 and the Wnt signalling pathway (*P* = 0.0003, Fig. 5B). As expected, SNHG20 knockdown markedly reduced the protein but not the mRNA level of β-catenin in U2932 and OCI-LY3 cells (*P* < 0.05, Fig. 5C and Supplementary Fig. 3). β-catenin mRNA expression was upregulated in DLBCL tissues compared to normal controls (Supplementary Fig. 4), as suggested by analysis via the GEPIA2 platform [21]. A previous study reported that an inhibitor of USP14 and UCHL5, b-AP15, inhibits DLBCL growth by suppressing the Wnt/β-catenin and TGFβ/Smad pathways [22]. In addition, β-catenin is recognized as a substrate of USP14 in mammalian cells [23]. The RIP assay confirmed the binding of SNHG20 to USP14 (*P* < 0.05, Supplementary Fig. 5). USP14 overexpression prominently increased the β-catenin level, and this increase was markedly attenuated by SNHG20 knockdown in U2932 and OCI-LY3 cells (*P* < 0.05, Fig. 5D and Supplementary Fig. 5). However, SNHG20 knockdown did not impact USP14 expression in DLBCL cells (Fig. 5D and Supplementary Fig. 5). Treatment with the proteasome inhibitor MG132 reversed SNHG20-induced β-catenin downregulation (*P* < 0.05, Fig. 6A). Moreover, SNHG20 knockdown decreased the half-life of β-catenin and increased the ubiquitination of β-catenin in DLBCL cells (Fig. 6B and C). From a mechanistic perspective, we confirmed that SNHG20 knockdown attenuated the interaction between USP14 and β-catenin (Fig. 6D). Thus, SNHG20 is required for USP14-mediated β-catenin protein stability in DLBCL cells.

### β-catenin knockdown abrogates SNHG20-facilitated DLBCL progression

**Fig. 7:**
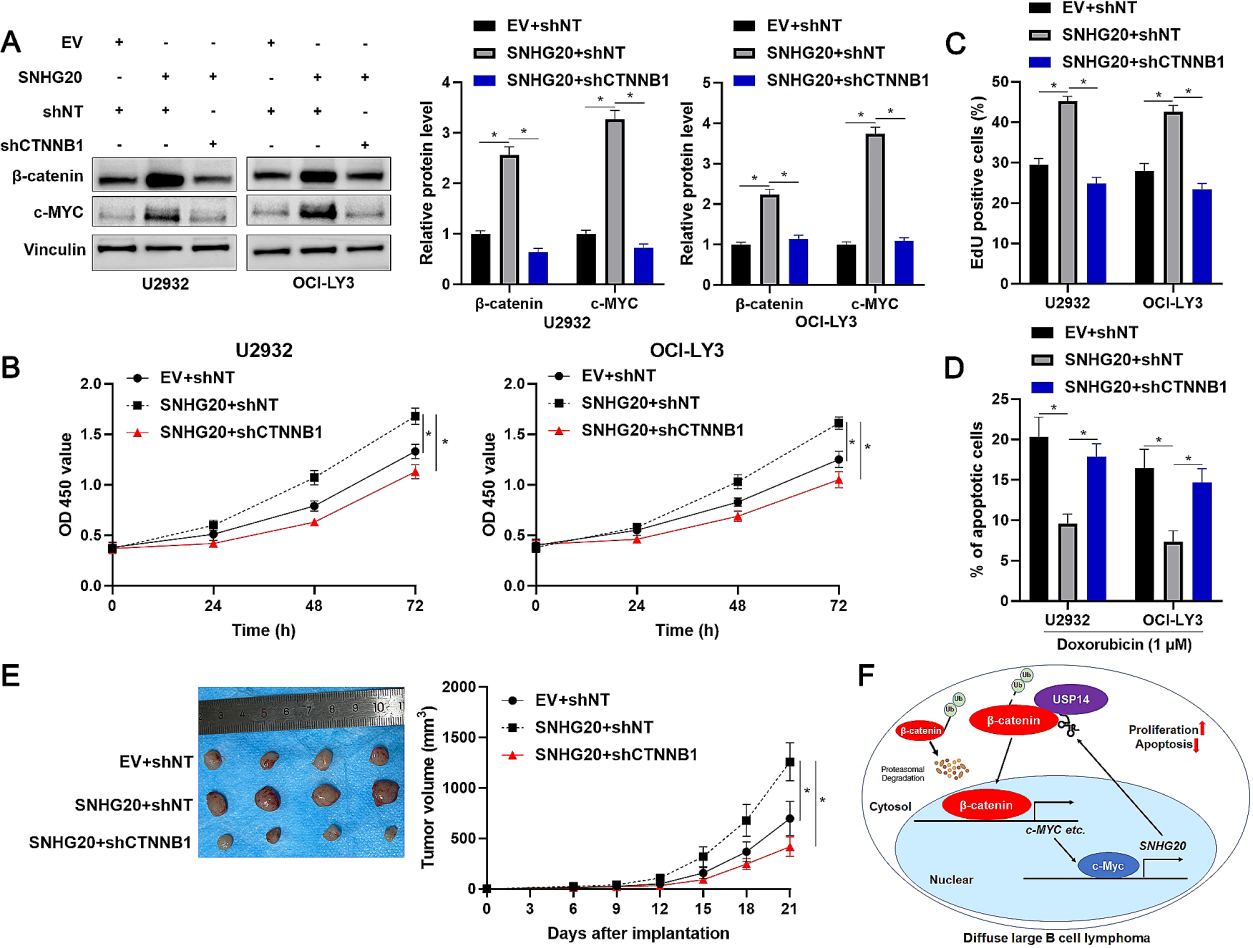
SNHG20-facilitated DLBCL cell proliferation and apoptosis resistance are attenuated by β-catenin knockdown. (**A**) U2932 and OCI-LY3 cells were transduced with the corresponding vectors and subjected to WB for measurement of β-catenin and c-MYC levels. (**B**) The viability of DLBCL cells transduced with the corresponding vectors was evaluated by using a CCK-8 assay. (**C**) The proliferation of DLBCL cells transduced with the corresponding vectors was examined by using an EdU incorporation assay. (**D**) Differences in apoptosis were evaluated using flow cytometry in DLBCL cells transduced with the corresponding vectors after 24 h of DOX treatment. (**E**) OCI-LY3 cells transduced with the corresponding vectors were subcutaneously injected into nude mice. Tumour volumes were compared to detect differences in tumour growth. (**F**) Schematic representation of the findings of the current study. *P < 0.05

To confirm the role of β-catenin in the SNHG20-mediated increase in DLBCL progression, β-catenin was knocked down in DLBCL cells with SNHG20 overexpression (*P* < 0.05, Fig. 7A). Interestingly, SNHG20 overexpression significantly increased the level of c-MYC in U2932 and OCI-LY3 cells, and the SNHG20-mediated increase in c-MYC expression was strongly attenuated by β-catenin knockdown (*P* < 0.05, Fig. 7A). As shown in Fig. 7B and C, β-catenin knockdown abolished the SNHG20-mediated increase in cell proliferation in U2932 and OCI-LY3 cells (*P* < 0.05). Twenty-four hours after DOX treatment, β-catenin silencing was found to significantly increase the SNHG20-mediated suppression of apoptosis in DLBCL cells (*P* < 0.05, Fig. 7D). The in vivo experiments confirmed that SNHG20 overexpression markedly promoted DLBCL growth and that this effect was attenuated by β-catenin knockdown (*P* < 0.05, Fig. 7E). Taken together, these findings indicated that SNHG20 promoted DLBCL progression by upregulating β-catenin.

## Discussion

In the last two decades, increasing attention has been focused on lncRNAs, which may play fundamental biological roles in the development of DLBCL. For instance, the lncRNA SNHG14 functions as a molecular sponge to bind miR-5590-3p, thereby increasing zinc finger E-box binding homeobox 1 (ZEB1) expression and accelerating the progression and immune evasion of DLBCL [24]. LncNBAT1 represses the transcriptional activity of signal transducer and activator of transcription 1 (STAT1) to attenuate apolipoprotein B mRNA editing enzyme catalytic subunit 3 A (APOBEC3A) expression, which mediates chemoresistance in DLBCL cells [25]. The lncRNA SNHG20 has been demonstrated to play oncogenic roles in many cancers, including oral squamous cell carcinoma [26], ovarian cancer [27], colorectal cancer (CRC) [28], cervical cancer [29], lung cancer [10], glioma [11] and hepatocellular carcinoma [30]. However, its role in haematological diseases, especially DLBCL, has never been studied. Our current study demonstrated that SNHG20 was overexpressed in DLBCL cell lines and tissues. Through in vitro experiments, we found that SNHG20 knockdown inhibited the proliferation and induced the apoptosis of DLBCL cells. However, the overexpression of SNHG20 had the opposite effects. Thus, SNHG20 was identified as a novel oncogenic driver in DLBCL.

The oncogene c-MYC plays vital roles in the progression of many human cancers through transcriptional upregulation to facilitate the malignant behaviours of cancer cells [31]. In addition to protein-coding genes, several lncRNAs are transcriptionally activated by c-MYC in human cancers. The lncRNA MNX1-AS1, which is upregulated by c-MYC, contributes to CRC progression by increasing Y-box-binding protein 1 (YB1) stability [32]. In DLBCL, c-MYC has been implicated in the transcriptional activation of the lncRNAs NEAT1 and FIRRE, which facilitate cancer cell proliferation. In this study, SNHG20 was identified as a novel target gene of c-MYC. C-MYC functions as a transcription factor of SNHG20 to positively regulate its expression in DLBCL. Therefore, our data provide new insight into the mechanism underlying c-MYC-driven DLBCL progression.

The subcellular localization of lncRNAs determines their functional mechanisms [33]. We found that SNHG20 was expressed mainly in the cytoplasm. Thus, SNHG20 may function as a scaffold or sponge for transcripts or proteins. Our subsequent KEGG pathway enrichment analysis suggested the potential regulatory role of SNHG20 in the Wnt signalling pathway. Interestingly, the protein but not the mRNA expression of β-catenin was prominently reduced by SNHG20 knockdown in DLBCL ells. A previous study reported that an inhibitor of USP14 and UCHL5, b-AP15, inhibits DLBCL growth by suppressing the Wnt/β-catenin and TGFβ/Smad pathways [22]. In addition, β-catenin is recognized as a substrate of USP14 in mammalian cells [23]. Our data confirmed that the USP14-promoted increase in β-catenin expression was abolished by SNHG20 knockdown. SNHG20 silencing increased the ubiquitination of β-catenin to accelerate its proteasomal degradation. Moreover, SNHG20 was found to be required for the interaction between USP40 and β-catenin. Therefore, our results suggest that SNHG20 is critical for USP14-mediated deubiquitylation of β-catenin in DLBCL cells. The regulatory role of SNHG20 in the Wnt/β-catenin signalling pathway has been reported in other human malignancies [34–37]. However, the regulatory mechanism is focused mainly on the function of SNHG20 as a ceRNA. Here, we first demonstrated that SNHG20 functioned as a scaffold for the interaction of USP14 with β-catenin and contributed to USP14-mediated β-catenin deubiquitination and stability. The Wnt/β-catenin signalling pathway is a critical oncogenic pathway for DLBCL progression and facilitates multiple malignant cellular behaviours and characteristics, including proliferation, apoptosis, and stemness [38–40]. In addition, c-MYC is a target gene of β-catenin [41]. Our data suggested the existence of a c-MYC/SNHG20/β-catenin positive feedback loop in DLBCL cells. Notably, we confirmed that β-catenin silencing attenuated SNHG20-facilitated DLBCL cell proliferation in vitro and in vivo.

In summary, we discovered that SNHG20 was overexpressed in DLBCL cells and tissues. Moreover, we demonstrated that SNHG20 was transcriptionally regulated by c-MYC in DLBCL. SNHG20 promoted DLBCL cell proliferation and apoptosis resistance by increasing the USP14-mediated deubiquitination and resulting stability of β-catenin. SNHG20 may be a novel promising therapeutic target for DLBCL in the future.

### Electronic supplementary material

Below is the link to the electronic supplementary material.

Supplementary Material 1

Supplementary Figure 1: SNHG20 increases DOX-induced apoptosis in DLBCL cells. (A) Changes Differences in apoptosis were evaluated using flow cytometry in U2932 and OCI-LY3 cells transduced with a nontargeting shRNA (shNT) or shRNAs against SNHG20 (shSNHG20-1 and shSNHG20-2) after 24 h of DOX (1 µM) treatment. (B) Changes Differences in apoptosis were evaluated using flow cytometry in U2932 and OCI-LY3 cells transduced with empty vector (EV) or a vector expressing SNHG20 (SNHG20) after 24 h of DOX (1 µM) treatment. *P < 0.05.

Supplementary Figure 2: the correlation between SNHG20 and c-MYC expression in DLBCL tissues. (A) Analysis of TCGA and GTEx data using the GEPIA2 platform indicated that c-MYC mRNA was more highly expressed in DLBCL tissues (T, red) than in normal tissues (N, grey). (B) A positive correlation between SNHG20 and c-MYC mRNA expression was detected in DLBCL tissues represented in the TCGA database.

Supplementary Figure 3: SNHG20 knockdown had no impact on the β-catenin mRNA level in DLBCL cells.

Supplementary Figure 4: β-catenin mRNA expression was upregulated in DLBCL tissues represented in the TCGA database. *P < 0.05.

Supplementary Figure 5: The RIP assay confirmed the binding of SNHG20 to USP14 in DLBCL cells. *P < 0.05.

Supplementary Figure 6: USP14 was overexpressed in DLBCL cells, and USP14 overexpression was not affected by SNHG20 knockdown. *P < 0.05.

## Data Availability

No datasets were generated or analysed during the current study.
