# Supplementary material for: C-MYC-activated lncRNA SNHG20 accelerates the proliferation of diffuse large B cell lymphoma via USP14-mediated deubiquitination of β-catenin
Source: Biol Direct. 2024 Jun 18;19:47. doi: 10.1186/s13062-024-00488-9 (PMC11184854; doi:10.1186/s13062-024-00488-9)
Supplement: Supplementary file 1 — Supplementary Material 1 [file 13062_2024_488_MOESM1_ESM.docx]

**Supplementary Table 1. The primers used in this study**

| **Gene** | **Forward primer (5’-3’)** | **Reverse primer (5’-3’)** |
| --- | --- | --- |
| SNHG20 | GCCAAGGTGACCACATACTCT | GCACTTGCTGGGTCGTTTG |
| β-catenin | AGCAATTTGTGGAGGGGGTC | AGCAGCTGCACAAACAATGG |
| SNHG20 for ChIP | GTTCCTGCTGCAGACCTCAT | CTGACCGCCTTTCATCACCT |
| β-actin | ATCCAGGCTGTGCTATCCCT | GGGCATACCCCTCGTAGATG |
| U6 | CTCGCTTCGGCAGCACA | AACGCTTCACGAATTTGCGT |
| GAPDH | GTCTCCTCTGACTTCAACAG  CG | ACCACCCTGTTGCTGTAGCC  AA |
