# Supplementary figures and images for: C-MYC-activated lncRNA SNHG20 accelerates the proliferation of diffuse large B cell lymphoma via USP14-mediated deubiquitination of β-catenin
Source: Biol Direct. 2024 Jun 18;19:47. doi: 10.1186/s13062-024-00488-9 (PMC11184854; doi:10.1186/s13062-024-00488-9)

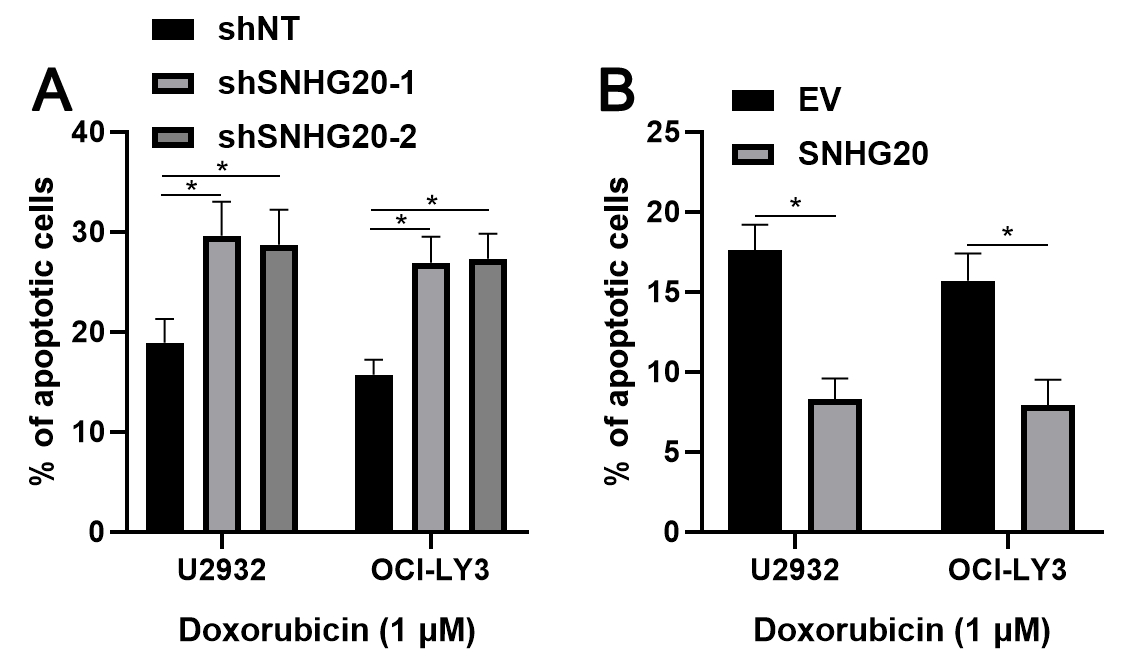

Supplement: Supplementary file 2 — Supplementary Figure 1: SNHG20 increases DOX-induced apoptosis in DLBCL cells. (A) Changes Differences in apoptosis were evaluated using flow cytometry in U2932 and OCI-LY3 cells transduced with a nontargeting shRNA (shNT) or shRNAs against SNHG20 (shSNHG20-1 and shSNHG20-2) after 24 h of DOX (1 µM) treatment. (B) Changes Differences in apoptosis were evaluated using flow cytometry in U2932 and OCI-LY3 cells transduced with empty vector (EV) or a vector expressing SNHG20 (SNHG20) after 24 h of DOX (1 µM) treatment. *P < 0.05. [file 13062_2024_488_MOESM2_ESM.tif]

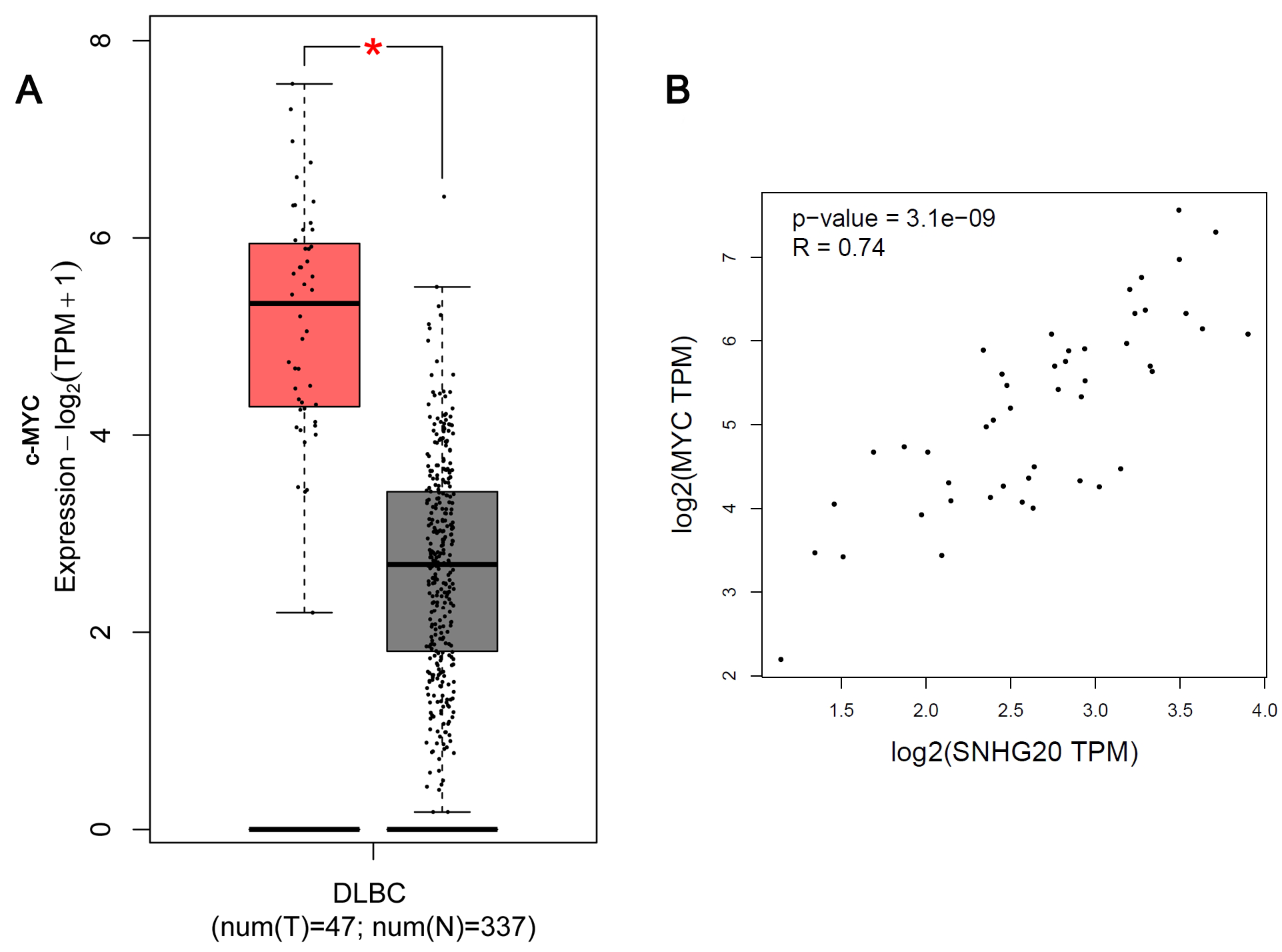

Supplement: Supplementary file 3 — Supplementary Figure 2: the correlation between SNHG20 and c-MYC expression in DLBCL tissues. (A) Analysis of TCGA and GTEx data using the GEPIA2 platform indicated that c-MYC mRNA was more highly expressed in DLBCL tissues (T, red) than in normal tissues (N, grey). (B) A positive correlation between SNHG20 and c-MYC mRNA expression was detected in DLBCL tissues represented in the TCGA database. [file 13062_2024_488_MOESM3_ESM.tif]

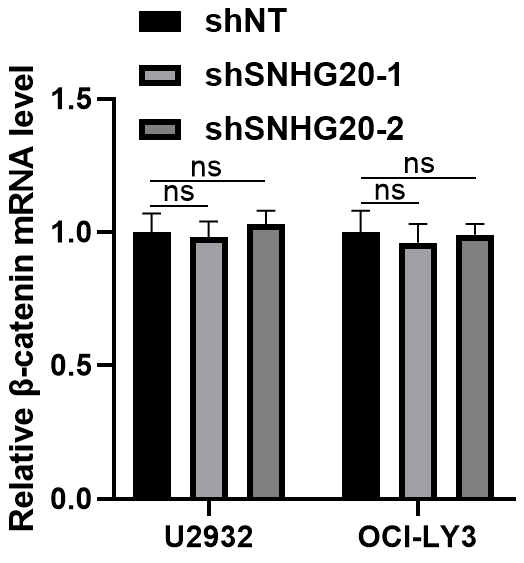

Supplement: Supplementary file 4 — Supplementary Figure 3: SNHG20 knockdown had no impact on the β-catenin mRNA level in DLBCL cells. [file 13062_2024_488_MOESM4_ESM.tif]

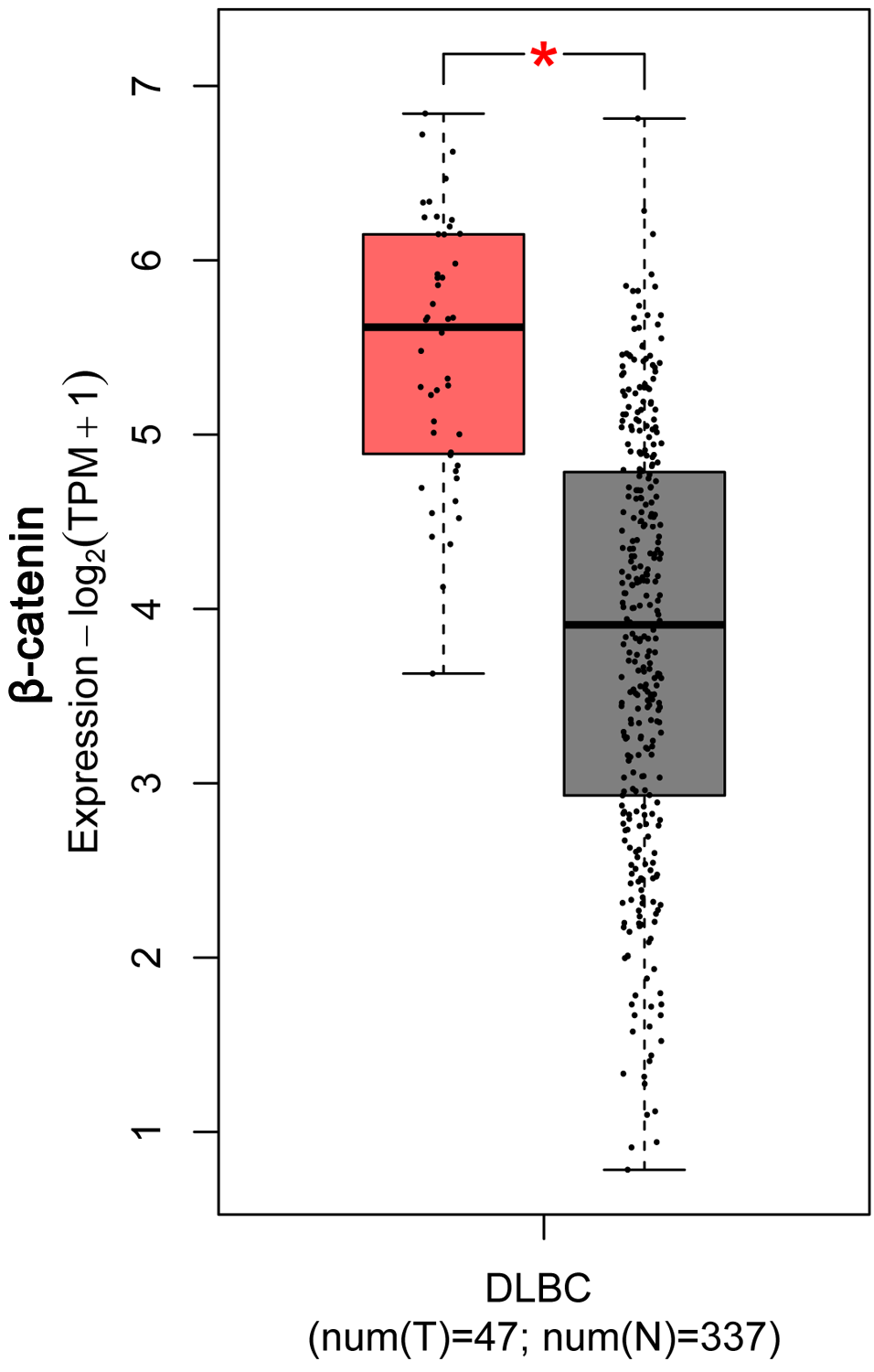

Supplement: Supplementary file 5 — Supplementary Figure 4: β-catenin mRNA expression was upregulated in DLBCL tissues represented in the TCGA database. *P < 0.05. [file 13062_2024_488_MOESM5_ESM.tif]

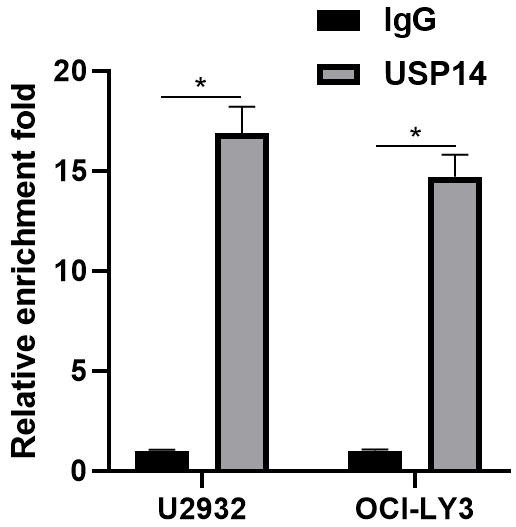

Supplement: Supplementary file 6 — Supplementary Figure 5: The RIP assay confirmed the binding of SNHG20 to USP14 in DLBCL cells. *P < 0.05. [file 13062_2024_488_MOESM6_ESM.tif]

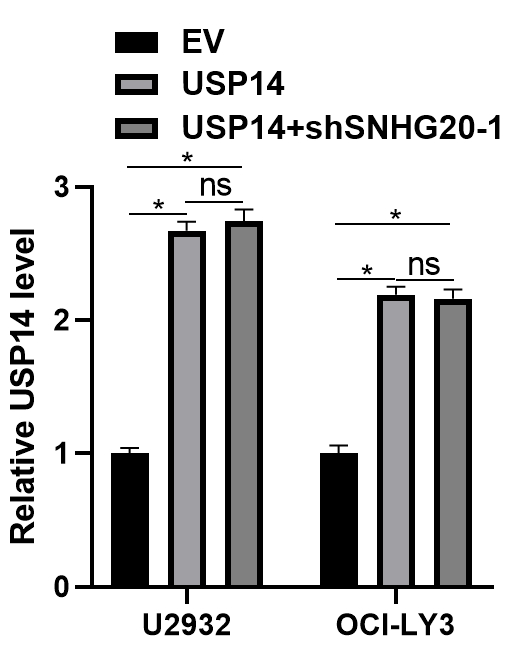

Supplement: Supplementary file 7 — Supplementary Figure 6: USP14 was overexpressed in DLBCL cells, and USP14 overexpression was not affected by SNHG20 knockdown. *P < 0.05. [file 13062_2024_488_MOESM7_ESM.tif]
